# 

**Published:** 1879-04

**Authors:** 


CONTENTS:

ARTICLE I.—Foul Breath. By J. T. Codman, D. D. S. .......... 529
ARTICLE II.—The Use of Chloroform in Dental Operations .......... 542
ARTICLE III.—Valedictory Address. By Rev. Dr. Alex. W. Weddell .......... 552
ARTICLE IV.—Class Address. By Frank B. Perry, D. D. S. .......... 561
ARTICLE V.—Compound Hare-Lip .......... 567

EDITORIAL, ETC.

Close of Volume, Etc .......... 569

OBITUARY.

Prof. John Hugh McQuillen, M. D., D. D. S. .......... 571

Prof. Philip H. Austen, M. D., D. D. S. .......... 576

